# Molecular characterization of *Clonorchis sinensis* secretory myoglobin: Delineating its role in anti-oxidative survival

**DOI:** 10.1186/1756-3305-7-250

**Published:** 2014-05-29

**Authors:** Mengyu Ren, Lei He, Yan Huang, Qiang Mao, Shan Li, Honglin Qu, Meng Bian, Pei Liang, Xueqing Chen, Jinsi Ling, Tingjing Chen, Chi Liang, Xiaoyun Wang, Xuerong Li, Xinbing Yu

**Affiliations:** 1 Department of Parasitology, Zhongshan School of Medicine, Sun Yat-sen University, 74 Zhongshan 2nd Road, Guangzhou, Guangdong 510080, China; 2 Key Laboratory for Tropical Diseases Control (Sun Yat-sen University), Ministry of Education, 74 Zhongshan 2nd Road, Guangzhou, Guangdong 510080, China; 3 Department of Pathogen Biology, Hainan Medical College, Haikou, Hainan 571199, China

**Keywords:** Myoglobin, *Clonorchis sinensis*, Point mutation, Hydrogen peroxide, RAW264.7, Peroxidase activity

## Abstract

**Background:**

Clonorchiasis is a globally important, neglected food-borne disease caused by *Clonorchis sinensis* (*C. sinensis*), and it is highly related to cholangiocarcinoma and hepatocellular carcinoma. Increased molecular evidence has strongly suggested that the adult worm of *C. sinensis* continuously releases excretory-secretory proteins (ESPs), which play important roles in the parasite-host interactions, to establish successful infection and ensure its own survival. Myoglobin, a hemoprotein, is present in high concentrations in trematodes and ESPs. To further understand the biological function of *Cs*Mb and its putative roles in the interactions of *C. sinensis* with its host, we explored the molecular characterization of *Cs*Mb in this paper.

**Methods:**

We expressed *Cs*Mb and its mutants in *E. coli* BL21 and identified its molecular characteristics using bioinformatics analysis and experimental approaches. Reverse transcription PCR analysis was used to measure myoglobin transcripts of *C. sinensis* with different culture conditions. The peroxidase activity of *Cs*Mb was confirmed by spectrophotometry. We co-cultured RAW264.7 cells with recombinant *Cs*Mb (r*Cs*Mb), and we then measured the production of hydrogen peroxide (H_2_O_2_) and nitric oxide (NO) in addition to the mRNA levels of inducible nitric oxide synthase (iNOS), Cu-Zn superoxide dismutase (SOD1) and Mn superoxide dismutase (SOD2) in activated RAW264.7 cells.

**Results:**

In the *in vitro* culture of adult worms, the transcripts of *Cs*Mb increased with the increase of oxygen content. Oxidative stress conditions induced by H_2_O_2_ increased the levels of *Cs*Mb transcripts in a dose-dependent manner. Furthermore, *Cs*Mb catalyzed oxidation reactions in the presence of H_2_O_2_, and amino acid 34 of *Cs*Mb played an essential role in its reaction with H_2_O_2_. In addition, *Cs*Mb significantly reduced H_2_O_2_ and NO levels in LPS-activated macrophages, and *Cs*Mb downregulated iNOS and SOD expression in activated macrophages.

**Conclusion:**

The present study is the first to investigate the peroxidase activity of *Cs*Mb. This investigation suggested that *C. sinensis* may decrease the redox activation of macrophages by *Cs*Mb expression to evade host immune responses. These studies contribute to a better understanding of the role of *Cs*Mb in the molecular mechanisms involved in ROS detoxification by *C. sinensis*.

## Background

Clonorchiasis is predominantly an endemic disease in countries and regions of eastern Asia. Approximately 35 million people are infected with this food-borne trematode, and more than 15 million people are infected with Clonorchiasis in China [1-3]. Chronic infections cause easy fatigue, abdominal pain, mechanical obstruction of the hepatobiliary duct, cholangiectasis and biliary stones [4]. Furthermore, *C. sinensis* has long been associated with cholangiocarcinoma in humans, and it has been classified as a group I carcinogen-metazoan parasite by the International Agency of Cancer Research of the World Health Organization [5]. Prior studies have demonstrated that adult liver flukes are able to survive in hosts for 20–25 years [6]. During long-term parasitism, the worm is continuously exposed to oxidizing molecules released by epithelial cells in the host ductal system and endogenous reactive oxygen species (ROS) generated by its own metabolic processes [7]. To ensure its long survival within the host bile ducts, the adult worm continuously releases bioactive molecules to cope with the cytopathic environments. The proteinaceous and nonproteinaceous components secreted by the parasites are referred to as excretory-secretory proteins (ESPs) [8]. The ESPs of *C. sinensis* (*Cs*ESPs) can cause histopathological changes, such as bile duct dilatation, inflammation and fibrosis as well as adenomatous proliferation of the biliary epithelium, which may be intimately associated with the formation of cholangitis, liver cirrhosis and cholangiocarcinoma (CCA).

Myoglobin (Mb), a hemoprotein, is present in high concentrations in trematodes and ESPs, and it may be implicated in host–parasite interactions. Most trematodes host cytoplasmic myoglobins, which are monomeric and approximately 17 kDa in size. Thus far, monomeric myoglobin/hemoglobin from the *Paramphistomum epiclitum* (*P. epiclitum*) is the best characterized trematode Mb, and it is monomeric and displays the major determinants of the typical globin fold [9-11]. The heme-ligand binding site displays a TyrB10/TyrE7 distal residue pair and a high oxygen affinity [12]. The crystal structure of *P. epiclitum* myoglobin shows that the heme distal site pocket residue, TyrB10, is engaged in hydrogen bonding to the iron-bound ligand [13].

The physiological roles of trematode Mbs are a matter of debate. Indeed, adult parasitic trematodes and nematodes, such as *Ascaris suum*, live mainly in a semi-anaerobic environment, and their Mbs display such high oxygen affinities that they cannot simply serve in O_2_ transport to the tissues. Therefore, other functions for these Mbs, such as oxygen scavenging, heme reserve for egg production, and NO dioxygenase, have been proposed [14-16]. Additionally, Mbs share many physical, spectroscopic and chemical similarities with authentic peroxidases. Thus, Mbs can act as a peroxidase and scavenge hydrogen peroxide (H_2_O_2_) [17,18], thereby protecting against oxidative damage [19]. Moreover, it has been reported that Dehaloperoxidase (DHP; discovered in the terebellid polychaete, Amphitrite ornata) is the first heme-containing globin with peroxidase activity. DHP retains an oxygen carrier function but also has the ability to degrade halophenol toxicants in its living environment [20]. Accordingly, Mbs might contribute to the defense against soluble hydroperoxide in catalase-deficient trematode species.

Here, we describe the molecular characteristics and biological function of CsMb as well as its putative role in the interaction of *C. sinensis* with its host. We investigated the transcriptional levels of *Cs*Mb in adult liver flukes under different culture conditions and observed the effects of recombinant *Cs*Mb (r*Cs*Mb) on RAW264.7 cell modulation. Based on these results, we proposed that *Cs*Mb functions as a component of the anti-oxidative survival strategy of *C. sinensis* in the host.

## Methods

### Ethics statement

All animals were housed in accordance with guidelines from the Association for the Assessment and Accreditation of Laboratory Animal Care (AAALAC). All protocols for animal infections were approved by the Institutional Review Board and conducted in the Institutional Animal Care and Use Committee (IACUC) of Sun Yat-sen University (permit number SCXK (Guangdong) 2009–2011).

### Preparation of parasites

*C. sinensis* metacercariae were collected from naturally infected freshwater fish (*Pseudorasbora parva*) in an ecological pool using pepsin digestion as previously described [21]. Sprague–Dawley rats were infected with metacercariae by a gavage needle (100 metacercariae per rat). Worms were harvested from the bile ducts after 12 weeks. Viable intact worms were collected under a dissecting microscope. Eggs of *C. sinensis* were obtained as described previously [22]. The intact living adults of *C. sinensis* were placed in serum-free DMEM medium (Gibco, USA) at 37°C for 1 h to ensure emptying of the guts. The worms were then incubated in the absence or presence of oxygen (1, 5 and 20%) at 37°C under 5% CO_2_ for 24 h or H_2_O_2_ (0–1.8 mM) at 37°C for 1 h (10 worms/group/3 ml of medium). Worms treated in DMEM medium at 37°C for 10 min were used as a negative control. Worms incubated at 37°C for 10 min in DMEM medium without H_2_O_2_ were included as negative controls. After incubation, the worms were washed three times with ice-chilled phosphate-buffered saline (PBS; pH 7.2) and were immediately used for the extraction of proteins and RNA. All procedures were repeated in triplicate.

### Bioinformatics analysis of *Cs*Mb

Homologous sequences were searched for using the Basic Local Alignment Search Tool X (BLASTx) program of the National Center for Biotechnology Information (NCBI), National Institutes of Health (Bethesda, Maryland). Sequence alignment with other previously reported Mbs (Sequence information shown in Additional file 1: Table S1) in the database was performed using the Clustal W (version 1.82) program of the European Bioinformatics Institute server (http://www.ebi.ac.uk/Tools/msa/clustalw2/). Signal peptides were predicted by SignalP (http://www.cbs.dtu.dk/services/SignalP/). Predicted motifs and secondary structures were obtained through PredictProtein (https://www.predictprotein.org/) provided by the Columbia University Bioinformatics Center (New York, USA). The Conserved Domain Database of NCBI was used to identify the protein families and domains. The tertiary structures of *Cs*Mb were simulated by the Discovery Studio 3.5 Client program and compared with *P. epiclitum* hemoglobin (Protein Data Bank id: 1KFR; 40% identity). The PHYLIP 3.67 program package was used for phylogenetic tree reconstructions, and divergence rates were calculated using the Jukes-Cantor-Thornton mode. The statistical significance of branching nodes was predicted by observing their frequencies in 1000 bootstrap trees using the Seqboot program.

### Preparation of recombinant *Cs*Mb, antiserum of recombinant *Cs*Mb and *Cs*ESPs

The expression and purification of recombinant *Cs*Mb (r*Cs*Mb) as well as the preparation of the antiserum of r*Cs*Mb and *Cs*ESPs are described in the additional file.

### Site-directed mutagenesis of *C. sinensis* myoglobin

Site-directed mutants of Y34A and Y68A were generated using the QuickChange II site-directed mutagenesis kit (Agilent, USA) using the primers listed in Table 1. All mutations were confirmed with DNA sequencing performed by the Meiji Biotechnology Company (Shanghai, China). The mutant proteins were expressed and purified as described in the additional file.

**Table 1:** Primers used in this study

**Primer**	**Sequence (5′ → 3′)**
Generation of recombinant *Cs*Mb protein	
*Cs*Mb-F	AGAGGATCCATGGCACCCCTATCAA
*Cs*Mb-R	CGTCTCGAGTTAGCCAAGAAAGCCG
Preparation of mutant *CsMb* proteins	
*Cs*Mb-*Y34A*-F	GAATTTGGAAAAGCAGTCGCCATGGCTCTGTTCTCAG
*Cs*Mb-*Y34A*-R	CTGAGAACAGAGCCATGGCGACTGCTTTTCCAAATTC
*Cs*Mb-*Y68A*-F	GAGGGGATTAAGTACGCCGGCCAGACCTTTGC
*Cs*Mb-*Y68A*-R	GCAAAGGTCTGGCCGGCGTACTTAATCCCCTC
Detection of *CsMb* transcripts via RT-PCR	
*Cs*Mb-RT-F	GCTGAACCCGTTGGTGAGTA
*Cs*Mb-RT-R	TCACTGGTAGAGATGAAAGGGC
*Cs*β-actin -RT-F	ACCGTGAGAAGATGACGCAGA
*Cs*β-actin -RT-R	GCCAAGTCCAAACGAAGAATT
Mouse specific primers	
iNOS-F	GCAATATAGGCTCATCCAG
iNOS-R	AACTCGCTCCAAGATTCC
Sod1-F	ATTACAGGATTAACTGAAGG
Sod1-R	CAATGATGGAATGCTCTC
Sod2-F	ACAAACCTGAGCCCTAAG
Sod2-R	CTCCCAGTTGATTACATTCC
β-actin -F	AACCGCGAGAAGATGACCCAGATCATGTTT
β-actin -R	AGCAGCCGTGGCCATCTCTTGCTCGAAGTC

### Immunohistochemical localization of *Cs*Mb in *C. sinensis* adults and metacercariae

Fresh adult worms and metacercariae of *C. sinensis* were fixed with 4% formaldehyde, embedded with paraffin wax, and sliced into 4-μm thick sections. After dewaxing and dehydration, the slides were blocked with goat serum overnight at 4°C, and the slides were then incubated with rat anti-r*Cs*Mb sera (1:100 in 0.1% PBS-T) at room temperature for 2 h. Serum from naïve rats was used as a negative control. The slides were washed twice and incubated with goat anti-rat IgG labeled with red fluorescent Cyanine dye 3 (Cy3, Proteintech; 1:400 in 0.1% PBS-T). Fluorescence microscopy (ZEISS Axio Imager Z1 fluorescent microscope, GER) was used to visualize the antibody staining.

### Peroxidase activity of r*Cs*Mb and mutants

The peroxidase activities of wild-type and mutant *C. sinensis* myoglobins were measured at 20°C in 50 mM sodium phosphate buffer (pH 7.0). All experiments were performed in duplicate for each experimental point. The steady state kinetic constants for the oxidation of guaiacol and 2,2’-azinobis(3-ethylbenzothiazoline-6-sulfonic acid) (ABTS) were obtained by measuring the initial rates while varying the substrate concentration. A Hanes plot of [S]/*v versus* [S] was used to estimate the V_max_ and K_m_ values. The formation rate of the guaiacol oxidation product was determined from the increase in the absorbance at 470 nm using a molar extinction coefficient of 3.8 × 10^3^ M^-1^ cm^-1^. The 1-ml final assay volume contained 1 μM r*Cs*Mb, 0.2 mM H_2_O_2_, and variable amounts of guaiacol (0.08 –2.5 mM). The formation of an ABTS cation radical was monitored at 730 nm, where the absorption of r*Cs*Mb was negligible. The absorption coefficient of the ABTS cation radical at 730 nm (ϵ_730_ = 1.4 × 10^4^ M^-1^ cm^-1^) was calculated from that at 415 nm (3.6 × 10^4^ M^-1^ cm^-1^). The reaction mixture contained 0.5 μM r*Cs*Mb, 0.2 mM H_2_O_2_, and 20 –300 mM ABTS.

### Treatment of RAW264.7 cells

RAW 264.7 macrophages were purchased from American Type Culture Collection (ATCC). RAW 264.7 cells were maintained in DMEM supplemented with 10% (v/v) fetal bovine serum (FBS), 100 U/ml penicillin and 100 μg/ml streptomycin at 37°C (5% CO_2_). Exponentially growing RAW264.7 cells were digested with 2.5 mg/ml trypsin and suspended in DMEM to a concentration of 2 × 10^5^ cells/ml. Subsequently, the cells were plated in 6-well flat-bottomed microculture plates (2 ml/well) and cultured at 37°C in a 5% CO_2_ atmosphere for 2 h. The cultures were washed to remove non-adherent cells and then incubated with 2 ml of DMEM supplemented with 10% (v/v) fetal bovine serum, 100 U/ml penicillin and 100 μg/ml streptomycin for an additional 20 h.

For the experiments, the following three groups of cells were used: control group, cells were activated with only PBS; LPS group, cells were activated with LPS and PBS; and r*Cs*Mb group, cells were activated with LPS and r*Cs*Mb. The culture medium of all groups was replaced with FBS-free DMEM for 30 min to allow cells to adjust. LPS (100 ng/ml) was then added to all groups, except the control group, to activate the macrophages. Purified r*Cs*Mb was added to each group at a final concentration of 5 μg/ml. Cells were further incubated for the desired experimental periods.

### Determination of NO and H_2_O_2_

The level of NO and H_2_O_2_ was analyzed by a Nitric Oxide Assay Kit and Hydrogen Peroxide Assay Kit (Beyotime Institute of Biotechnology, China). The absorbance was measured at 540 nm using a microplate reader (Biorad Benchmark, USA).

### RNA extraction and complementary DNA (cDNA) synthesis

Total RNA from adult worms, excysted metacercariae, metacercariae, and eggs was extracted in Trizol reagent (Invitrogen) according to the protocol recommended by the manufacturer. RNA concentration and quality were detected by a nucleic acid/protein analyzer (Beckman Coulter, USA) and agarose gel electrophoresis, respectively. For reverse transcription, the reaction was performed in a final volume of 20 μl containing 2 μg of total RNA, 2.5 μM oligo dT primer, and five units of avian myeloblastosis virus (AMV) reverse transcriptase (TaKaRa, Japan). The reaction mixture was then incubated for 1 h at 42°C and heated for 5 min at 95°C.

### Real-time quantitative reverse transcription-PCR (real-time qRT-PCR)

To analyze the mRNA transcription levels of CsMb at different developmental stages of C. sinensis, PCR was performed using the cDNA templates from different developmental stages of C. sinensis, which contained 10 μl of 2 × SYBR Premix Ex Taq (TaKaRa, Japan), 2 μl of diluted cDNA template, 7.2 μl of PCR-grade water, and 0.4 μl of each 10 μM primer. The PCR conditions were as follows: 95°C for 30 s followed by 40 cycles of 95°C for 5 s and 60°C for 20 s with a 0.1°C/s incremental increase from 60 to 95°C. *Cs*Mb primers were specifically designed by Premier 5.0, we did test all primers specific efficiency by Primer-BLAST. *C. sinensis* β-actin (GenBank accession number EU109284) was used as an internal control. All primers used in this study are listed in Table 1. The relative quantification analysis was performed by calculating the values of 2^-ΔΔCT^. All experiments were performed in duplicate for each experimental point.

### Statistical analysis

All experiments were repeated at least three times, and the data were analyzed by one-way analysis of variance (ANOVA) and the Students-Newmann-Keuls (SNK) test using the SPSS software package 17.0. Experimental values were obtained from three independent experiments with a similar pattern and expressed as the means ± standard deviation (SD). P-values less than 0.05 were considered significant.

## Results

### Bioinformatics analysis

**Figure 1:**
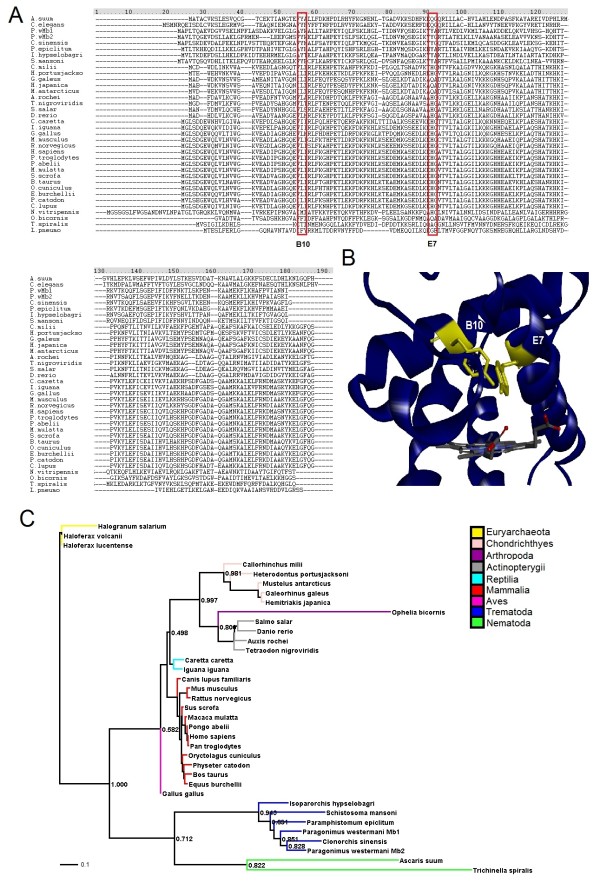
**Sequences and structure comparisons between *Clonorchis sinensis *myoglobin ( *Cs *Mb) and its orthologues. (A)** The amino acid sequence of *Cs*Mb was aligned with those of related proteins, which were retrieved from the GenBank database by BLAST searches (Sequence information shown in Additional file 1: Table S1). The red boxes indicate active tyrosine. **(B)** The tertiary structure of *Cs*Mb was simulated by the Discovery Studio 3.5 Client program and compared with *P. epiclitum* hemoglobin (Protein Data Bank id: 1KFR; 40% identity). Regions around the active site are shown in yellow indicating the positions of critical amino acids (red boxes in panel **A**). **(C)** Phylogenetic analysis. The majority-role consensus tree was derived from a neighbor-joining tree of the amino acid alignment, which was constructed by the PHYLIP program. The homologues from euryarchaeota were included in the root of the tree. Arabic numerals at branching nodes indicate their percentages of appearance in 1000 bootstrap replicates.

The *Cs*Mb gene was identified from the *C. sinensis* genome [23]. Analysis of the presence of a secretory signal peptide indicated that *Cs*Mb had no signal peptide sequence and that it had a value higher than the threshold value (0.5) for the possibility of non-classical secretion. Sequence alignment revealed that *Cs*Mb contained the characteristic tyrosyl residue substitution at the helical positions of B10 and E7 (distal) of trematode Mbs (Figure 1A), which distinguished the Mbs of trematode from other organisms. PredictProtein indicated the presence of 8 helices in *Cs*Mb, which are fundamental to Mb proteins. The comparison around the active site is shown in Figure 1B.

### Phylogenetic position of *Cs*Mb

Based on the BLAST search results, Mb amino acid sequences of 37 species were selected to examine the phylogenetic position of *Cs*Mb. The neighbor-joining method in PHYLIP clearly separated these proteins into distinct clades according to the taxonomical positions of the donors, such as euryarchaeota, nematoda, trematoda, chordata and mammalia (a major rule tree is presented in Figure 1C). The phylogenetic tree indicated that *Cs*Mb had high homology to trematode Mbs. Among the trematoda, the two Mbs of *P. westermani* were related to *Cs*Mb. As expected, Mbs from vertebrates and trematoda formed their respective groups. *Sperm whale* Mb, an extensively studied and well-characterized Mb, was included as a typical representative of mammalian Mbs that are clearly different from trematoda Mbs in the primary structure. Euryarchaeota Mbs were used to root the tree.

### Immunolocalization of *Cs*Mb in the adult worm and metacercaria of *C. sinensis*

**Figure 2:**
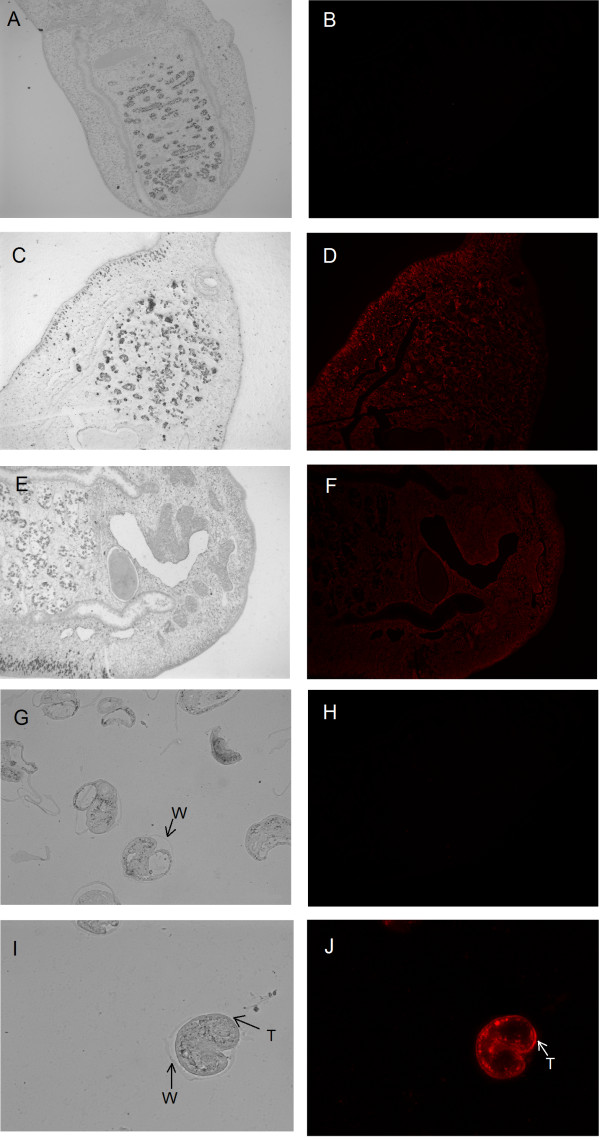
**Immunolocalization of CsMb in adult worm and metacercaria of *C. sinensis*.** Rat anti-r*Cs*Mb serum was used as the primary antibody and red fluorescent Cy3-labeled goat anti-rat IgG as the secondary antibody. Slides were observed under white light (panel **A**, **C**, **E**, **G**, and **I**) or under a fluorescence microscope (panel **B**, **D**, **F**, **H**, and **J**). No specific fluorescence was observed in panel **B** or **H**, which was probed with serum from rats immunized with PBS as a negative control. Intensive reddish-orange fluorescent signals were observed in the subtegumental and mesenchymal tissues of the adult worm (panel **D** and **F**; ×50) as well as the vitellarium of the metacercaria (panel **J**; ×200). Scattered fluorescent signals were detected in the tegument of the metacercaria. T, tegument. W, cyst wall.

The analysis of immunofluorescence localization in *C. sinensis* adults using rat anti-r*Cs*Mb serum showed intense intracellular fluorescence in the subtegumental and mesenchymal tissues. In the metacercaria, the fluorescence was localized in the tegument and vitellarium (Figure 2D, F and J). By comparison, no specific fluorescence was detected in either the adult worm or metacercaria when treated with serum from naïve rats (Figure 2B and H).

### Transcriptional levels of Mb in *C. sinensis* incubated in different conditions

**Figure 3:**
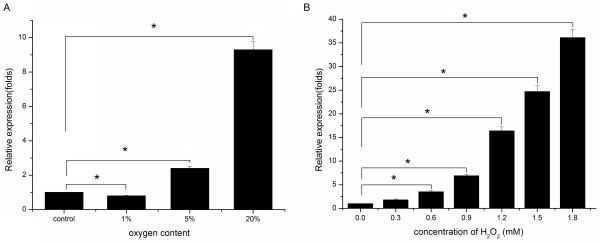
**Transcriptional level of Mb in *C. sinensis *incubated in different oxygen contents. (A)** The fold increase was calculated by comparing intensities between experimental and control groups. The *Cs*Mb transcripts were much higher in the 20% oxygen group than in the 1% oxygen group. The transcriptional level of *Cs*Mb in the 5% oxygen group was 3-fold higher than that in the 1% oxygen group. **(B)** Changes of Mb transcript levels in *C. sinensis* caused by oxidative chemicals. The worms were stimulated with H_2_O_2_ (0–1.8 mM) for 1 h at 37 °C. *Cs*Mb transcript amounts were significantly increased at H_2_O_2_ concentrations greater than 0.6 mM. (*p < 0.01).

*Cs*Mb mRNA levels were detected by real time-PCR when the parasites were cultured in three different conditions. Interestingly, the transcription level of *Cs*Mb in 20% oxygen was the highest, and the transcription level of *Cs*Mb in 1% oxygen was the lowest (Figure 3A). We assessed the induction profile of *Cs*Mb upon stimulation with oxidative chemicals (H_2_O_2_). The worms were stimulated with H_2_O_2_ (0–1.8 mM) at 37°C for 1 h. Oxidative stress conditions created by H_2_O_2_ increased the mRNA level of *Cs*Mb in a dose-dependent manner (Figure 3B). There was no corresponding change at the transcription level of β-actin, so we know the response is specific.

### Peroxidase activity of r*Cs*Mb and mutants

**Figure 4:**
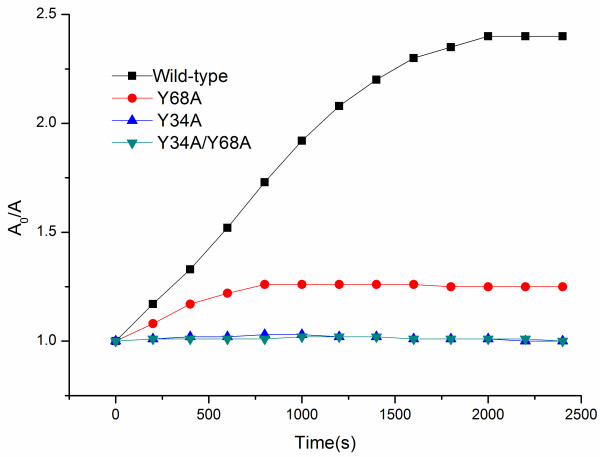
**Peroxidase activity of r *Cs *Mb and mutants evaluated by spectrophotometry**. Reactions were performed with H_2_O_2_ (20 μM) and oxygenated r*Cs*Mb solutions at 20 °C and pH 7.0. The A_0_/A ratio is the initial absorbance and final absorbance at 407 nm. All mutants containing Y34A did not show H_2_O_2_ reactivity, and only Y68A exhibited lower reactivity than that of wild-type *Cs*Mb.

The residues at sites 34 and 68 of the r*Cs*Mb mutants are described in Additional file 1: Table S2. The V_max_ and K_m_ values for the peroxidase activity are summarized in Table 2. Wild-type *Cs*Mb exhibited V_max_ values 6-fold higher than those of the Y68A mutant both in the guaiacol and ABTS oxidation assays, and no significant activities for the Y34A and Y34A/Y68A mutants were observed. From the *Cs*Mb mutant reactions, we found that the tyrosyl at residue 34 played a critical role to activate H_2_O_2_. As shown in Table 2, removal of Tyr34 (Y34A mutants) showed no reactivity. To better illustrate the dual tyrosines at positions 34 and 68 accelerating the peroxidase activity, we plotted the time course for the reactions of different r*Cs*Mbs mutants with H_2_O_2_ according to the changes of absorbance at 407 nm. The kinetic trace obeyed pseudo first-order kinetics in the incubation with 25 mM H_2_O_2_ (Figure 4).

**Table 2:** **Rate constants of reactions between different mutants and H**_**2**_**O**_**2**_

***Cs*** **Mb**	**Guaiacol**		**ABTS**	
	**V**_**max**_	**K**_**m**_	**V**_**max**_	**K**_**m**_
Wild-type	46	81	167	66
Y34A	N.D. ^*^	N.D. ^*^	N.D. ^*^	N.D. ^*^
Y68A	7.1	590	32	83
Y34A/Y68A	N.D. ^*^	N.D. ^*^	N.D. ^*^	N.D. ^*^

Reactions were performed with H_2_O_2_ (~20 μM) and oxygenated rCsMb solutions at 20 °C and pH 7.0. The units are as follows: V_max_, nmol of product/nmol of rCsMb /min; and K_m_, mM.

^*^Reaction rates were too slow to measure.

### Production of H_2_O_2_ and NO in activated RAW264.7 cells

**Figure 5:**
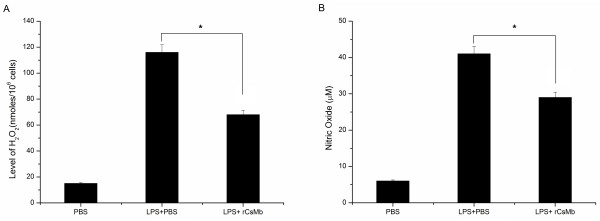
**Production of H**_**2**_**O**_**2 **_**and NO in activated RAW264.7 cells in various conditions.** In the respective experimental conditions, the H_2_O_2_ level was measured after 2 h **(panel A)**, and the NO level was measured after 24 h **(panel B)**. LPS (100 ng/ml) was used as a positive control. The levels of H_2_O_2_ and NO were significantly decreased in r*Cs*Mb-treated cells compared to the levels of cells treated with LPS + PBS. * indicates *p* < 0.01.

Respiratory burst activity of macrophages in terms of H_2_O_2_ and NO levels were measured after 2 h and 24 h, respectively. The r*Cs*Mb-treated (LPS + r*Cs*Mb) cells showed lower levels of NO and H_2_O_2_ (P < 0.05, Figure 5) when compared to the LPS-activated (LPS + PBS) cells.

### Quantitation of mRNA levels of inducible nitric oxide synthase (iNOS), Cu-Zn superoxide dismutase (SOD1) and Mn superoxide dismutase (SOD2)

**Figure 6:**
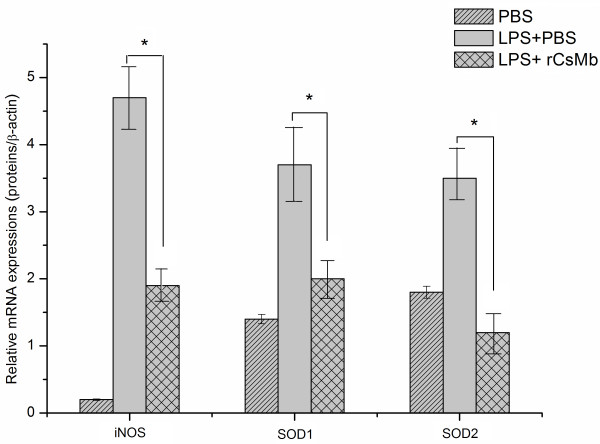
**mRNA levels of various genes involved in the oxidative burst in RAW264.7 cells.** The relative mRNA level is represented by the ratio of mRNAs to β-actin. The mRNA levels were significantly downregulated in cells treated with r*Cs*Mb compared the levels of cells treated with LPS + PBS. (*p < 0.01).

After a 24 h incubation in the respective experimental conditions, the mRNA levels of iNOS, SOD1 and SOD2 were measured (Figure 6). Compared to the LPS + PBS or LPS + r*Cs*Mb cells, the mRNA levels of iNOS, SOD1 and SOD2 were significantly decreased in r*Cs*Mb-treated cells.

## Discussion

The aim of this study was to evaluate the phylogenetic positions and functional characterization of *Cs*Mb as well as to address its role in the pathogenesis of Clonorchiasis. The expression of *Cs*Mb was highly inducible in response to exogenously introduced H_2_O_2_. Our results showed that *Cs*Mb has peroxidase activity and may be important in the detoxification against H_2_O_2_ and other ROS.

It is widely accepted that Mbs serve as oxygen stores rather than oxygen transporters or as oxygen sensors in some cases, and they are found in bacteria, plants, protozoans and invertebrate nerve systems. Bioinformatics analysis showed that *Cs*Mb comprised a globin family profile domain and heme-binding site. Although there was low amino acid sequence homology between the distantly related globins and the amino acid identity between human and *C. sinensis* myoglobins was only approximately 29%, the Mbs were found to have a similar three-dimensional structure (the so-called myoglobin fold) (Figure 1). Our phylogenetic analysis showed that the vertebrate groups of Mbs were more closely related to each other and that the invertebrate globins were more divergent. As reported elsewhere, nonvertebrate globins show higher variability in their primary and tertiary structures, which might reflect their adaptations to specific functions compared with their vertebrate homologs [24]. trMbs are the most primitive animal globins [25] as indicated by the phylogenetic analysis of their primary structure. Because trMbs have such a high oxygen affinity and low rate of dioxygen dissociation, it seems unlikely that the function of trMbs is oxygen delivery.

Our study showed that *Cs*Mb was transcribed in four developmental stages of *C. sinensis*, including adult worms, metacercariae, excysted metacercariae and eggs (Additional file 1: Figure S1). Quantitative real-time PCR analysis revealed that the transcription level of *Cs*Mb mRNA was much higher in the adult stage than in the other stages, which may have been due to *Cs*Mb playing an important role in long-term survival of the adult worms. Moreover, immunolocalization results showed that *Cs*Mb was extensively distributed intracellularly in the subtegumental and mesenchymal tissues of adult worms, and these results also showed that *Cs*Mb was localized in the teguments of metacercariae (Figure 2). A previous study on the expressed sequence tag analysis of *C. sinensis* adults discovered that *Cs*Mb is one of the most abundant proteins in adult worms [26]. Moreover, r*Cs*Mb can be probed by *C. sinensis*-infected rat sera and ESP-immunized rat sera. ESPs can be probed with rat anti-r*Cs*Mb sera, resulting in a single band with a MW of approximately 17 kDa. In the present study, western blotting analysis confirmed that *Cs*Mb was a molecular component of CsESPs (Additional file 1: Figure S2). As a food-borne parasite, *C. Sinensis* adult worms live in the bile ducts of the host, and the worm releases ESPs. Thus, *Cs*Mb exists in sustained parasitism circumstances and participates in the interaction between the host and parasite.

We also cultured the adult worms in different concentrations of oxygen. The expression of *Cs*Mb gene was analyzed by qRT-PCR, which showed that *Cs*Mb expression was upregulated after 24 h. The 5 and 20% oxygen groups showed 2.1- and 5.4-fold increases, respectively, compared to the control group. In contrast, *Cs*Mb gene expression in the 1% oxygen group was lower than that of the control group. These results demonstrated that *Cs*Mb may be involved in aerobic metabolism and in the oxygen reservoir of adult worms. Moreover, these results showed that the existence of exogenous H_2_O_2_ significantly increased *Cs*Mb transcripts of adult worms in a dose-dependent manner. Therefore, the expression of *Cs*Mb was highly inducible in response to exogenously introduced H_2_O_2_, and *Cs*Mb may exert its important role in response to exogenous stimulus.

Based on the transcriptional levels of *Cs*Mb under different culture conditions, we inferred that *Cs*Mb may have peroxidase activity in addition to its role in the transportation and storage of oxygen. An analysis of the amino acid sequences also showed that *Cs*Mb contained a TyrB10/TyrE7 distal residue pair, which is a characteristic sequence signature of trematode globins. Tyrosine is present at the B10 and E7 positions in trematoda instead of the more common Leu (B10) and His (E7) positions in other species. The E7 and B10 positions are key amino acids of the heme pocket. In this work, we found that *Cs*Mb had a specific peroxidase activity and that the tyrosine at residue 34 played a critical role in the reaction with H_2_O_2_. Replacing Tyr34 with Ala in *Cs*Mb resulted in no reaction to H_2_O_2_. Our finding suggested that *Cs*Mb is of great importance in the detoxification of H_2_O_2_ and other ROS. A significant H_2_O_2_ interaction with *Cs*Mb may question the viewpoint that a series of antioxidant enzymes, such as GSH S-transferase [27], Cys peroxiredoxins [28] and GSH peroxidase [29], participate in the removal of these detrimental oxidants. These enzymes are mainly responsible for dismutation of host oxidative stress to protect parasitic proteins, DNA and lipids from oxidative damage. Although these antioxidant enzymes have a higher affinity for H_2_O_2_ than *Cs*Mb, *Cs*Mb exists extensively and abundantly. Because decreased H_2_O_2_ levels eventually increase parasite survival and growth [30], *Cs*Mb may be an essential component of the main enzymatic scavenger for ROS in *C. sinensis*.

This study also showed that r*Cs*Mb decreased redox activation in a human macrophage cell line. In *C. sinensis* infection, reactive oxygen and nitrogen species play crucial roles in the initial events of parasite killing and elimination from the host [31]. To establish infection and ensure survival, *C. sinensis* has developed sophisticated strategies to evade the host immune responses. Despite a wealth of gained crucial information, these strategies still remain poorly understood. LPS is a potent activator of macrophage responses leading to the induction of various inflammatory mediators, such as reactive oxygen species, nitrogen species and inflammatory cytokines. We treated LPS-stimulated RAW264.7 cells with r*Cs*Mb, and we found that r*Cs*Mb effectively reduced H_2_O_2_ and NO levels in LPS-activated cells as well as the expression levels of iNOS and SODs. Because decreased H_2_O_2_ levels eventually augment parasite survival, it is likely that *C. sinensis* silences macrophages by secreting *Cs*Mb to suppress host immune responses. The results clearly showed that *Cs*Mb may have immunomodulatory functions.

The purpose of a host oxidative assault is to eliminate parasitic invasion, and it is also a critical link for various inflammatory responses of the host [32]. Parasites can regulate the host immune responses to maintain the parasitism for a prolonged period, but the molecules governing this process are not yet identified. Parasitic products interfere with lymphocytes and their products, such as antibodies, resulting in a modified immune response. *Ancylostoma caninum* produces factors, such as neutrophil inhibitory factor (NIF), capable of inhibiting the neutrophil-endothelium adhesion [33]. Our present study indicated that *Cs*Mb in adult worms not only acts to store oxygen but can also counter oxidative assaults. *Cs*Mb may take part in the anti-oxidative survival strategy of *C. sinensis* in the host.

## Conclusion

In conclusion, our data demonstrated that *in vitro* culture of adult worms with exogenous H_2_O_2_ increases the level of *Cs*Mb transcripts. The increased *Cs*Mb expression may potentially act as an antioxidant enzyme and may be involved in the host immune response. Thus, the reduced expression levels of iNOS and SODs as well as the low levels of NO and H_2_O_2_ in r*Cs*Mb-treated RAW264.7 cells may contribute to the control of the redox activation of macrophages during *C. sinensis* infection. The peroxidase activity of *Cs*Mb and decrease of redox activation in human macrophages may contribute to the establishment and maintenance of the anti-oxidative survival strategy of *C. sinensis* in the host. A detailed study is required to fully understand the role of *Cs*Mb in the pathogenesis of *C. sinensis* for effective control of Clonorchiasis worldwide.

## Competing interests

The authors declare that we have no competing interests.

## Authors’ contributions

MYR, LH, YH, XYW and XBY conceived and designed the experiments; MYR, LH, MB, QM, SL, HLQ, PL, JSL, TJC, CL performed the experiments; MYR, LH analyzed the data; MYR, LH, XRL and YH wrote the manuscript; All authors read and approved the final manuscript.

## Supplementary Material

Additional file 1: Figure S1 Quantitative real-time PCR analysis of CsMb at different life cycle stages of *C. sinensis*. **Figure S2**. Determination of ***Cs*** Mb as a component of ***C. Sinensis*** ESPs. **Table S1**. Sequences information of homologues from other species **Table S2**. Residues at site 34 and 68 of wild-type CsMb and different mutants. Click here for file
