# Supplementary material for: Molecular characterization of Clonorchis sinensis secretory myoglobin: Delineating its role in anti-oxidative survival
Source: Parasit Vectors. 2014 May 29;7:250. doi: 10.1186/1756-3305-7-250 (PMC4057808; doi:10.1186/1756-3305-7-250)
Supplement: Additional file 1: Figure S1 — Quantitative real-time PCR analysis of CsMb at different life cycle stages of C. sinensis. Figure S2. Determination of Cs Mb as a component of C. Sinensis ESPs. Table S1. Sequences information of homologues from other species Table S2. Residues at site 34 and 68 of wild-type CsMb and different mutants. [file 1756-3305-7-250-S1.docx]

**Supplementary materials and methods**

**Recombinant protein expression and purification of *Cs*Mb**

The gene of *Cs*Mb was amplified from our complementary DNA (cDNA) plasmid library of adult *C. sinensis* by polymerase chain reaction (PCR) using primers containing restriction sites for *Bam*HI and *Xho* I (Table 1). The PCR products were cloned into a prokaryotic expression vector pET28a (+) (Novagen, USA). The nucleotide sequence of the recombinant plasmids were confirmed by DNA sequencing, after which the constructs were, transformed to *E. coli* BL21 (DE3) competent cells. The r*Cs*Mb protein was expressed in Luria–Bertani medium at 37℃. When the cell density reached an OD600 of about 0.8, the protein expression was induced with 1.0 mM isopropyl-β-D-thiogalactopyranoside (IPTG) for 5 h at 37℃. *E. coli* cells were harvested, sonicated and the r*Cs*Mb proteins were purified by nickel–nitrilotriacetic acid (Ni–NTA) agarose chromatography (Qiagen, USA). The final concentration of purified rCsMb was determined by bicinchoninic acid (BCA) protein assay kit (Novagen, USA). The molecular weights and purity of the proteins were examined by 12% SDS–PAGE under reducing conditions. The purified recombinant proteins were stored at −80 °C for later use.

**Preparation of antiserum of recombinant *Cs*Mb and *Cs*ESPs**

Sprague–Dawley rats were purchased from animal center of Sun Yat-sen University. The r*Cs*Mb or *Cs*ESPs (50 μg of protein for each rat) was emulsified with equivalent complete Freund adjuvant and subcutaneously immunized. Subsequently each rat was given 25 μg of proteins for 3 booster injections at 2-week interval. Antiserum was collected at the 8^th^-weeks. The antibody titer was determined by enzyme-linked immunosorbent assay (ELISA).

**
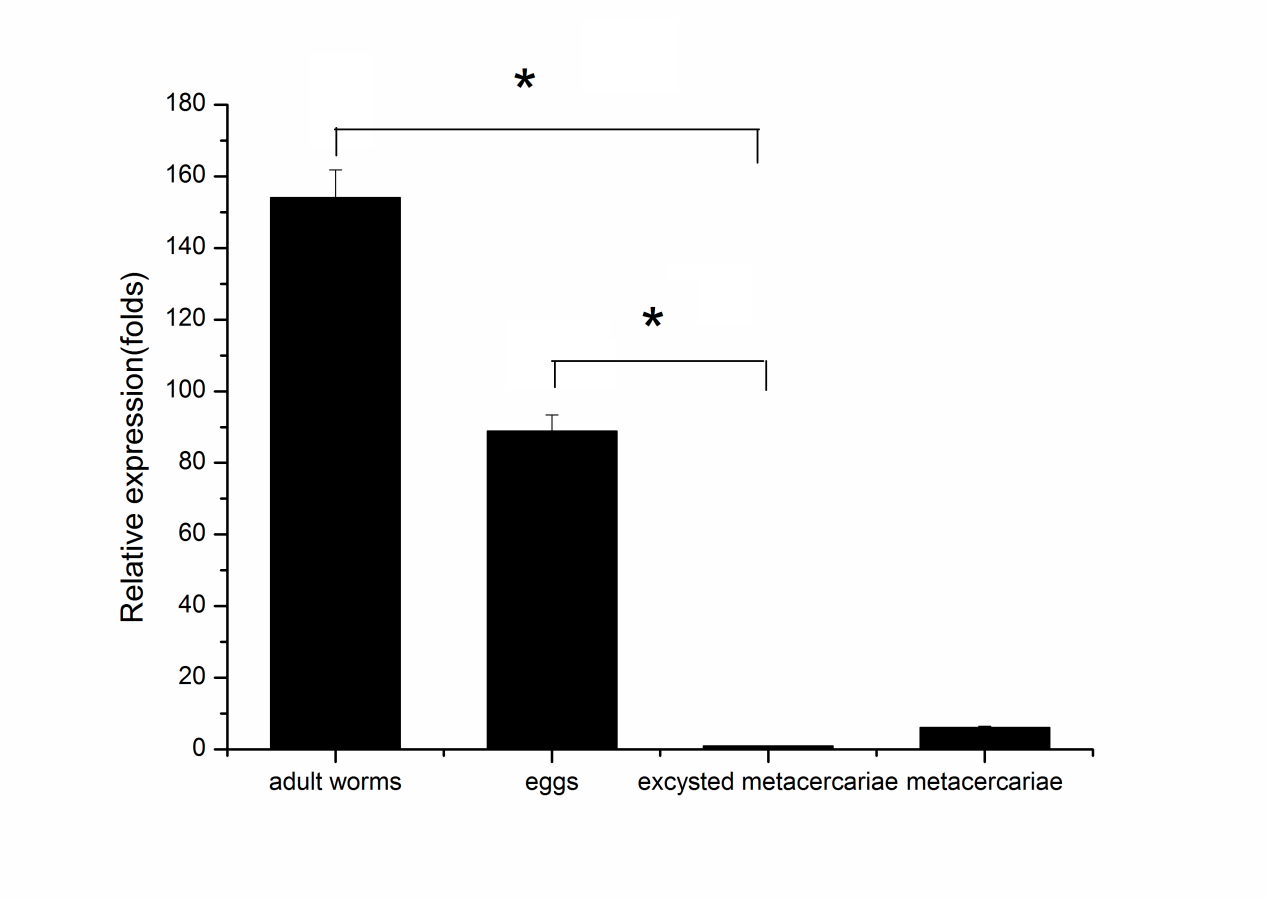
**

**Fig. S1**

**Quantitative real-time PCR analysis of *Cs*Mb at different life cycle stages of *C. sinensis*.** The transcripts of *Cs*Mb were detected in four developmental stages of *C. sinensis* including adult worms, metacercariae, excysted metacercariae and eggs. Transcription of excysted metacercariae was set as a calibrator to measure the relative mRNA levels of *Cs*Mb in other stages. The transcription levels of *Cs*Mb at life stages of adult worm, metacercaria, excysted metacercariae and eggs are analyzed by means of the 2^−ΔΔCT^ ratio, with *Cs*β-actin serving as the internal standard. *Cs*Mb showed a significantly higher transcription level at the stage of adult worm than those of excysted metacercariae (154.1-fold; *p* < 0.01), metacercariae (24.5-fold; *p* < 0.01), and eggs (1.7-fold; *p* < 0.01). Transcripts of *Cs*Mb were much higher in eggs than in excysted metacercariae (88.9-fold; *p* < 0.01) and metacercariae (14.1-fold; *p* < 0.01). * indicates *p* < 0.01. No difference was detected between adult worm and eggs (*p* > 0.05)

**

**

**Fig. S2**

**Determination of CsMb as a component of C. Sinensis ESPs.** Recombinant *Cs*Mb was probed with anti-His tag monoclonal antibody, rat anti-r*Cs*Mb serum, or serum from *C. sinensis* infected rat by western blotting (lane 1, lane 2 and lane 3). Rat anti-*Cs*ESP serum could specifically recognize r*Cs*Mb (lane 4). In addition, *Cs*ESPs was probed with rat anti-r*Cs*Mb serum at 18kDa (lane 5). Either r*Cs*Mb or *Cs*ESPs could not be probed with naive rat serum (lane 6, lane 7).

**Table S1: Sequences information of homologues from other species**

| Organism | Common name | Abbreviation | Accession no. |
| --- | --- | --- | --- |
| *Auxis rochei* | bullet tuna | *A. rochei* | BAD23846.1 |
| *Ascaris suum* | Large roundworm of pigs | *A.suum* | P49672.1 |
| *Bos taurus* | cattle | *B.taurus* | NP_776306.1 |
| *Callorhinchus milii* | Elephant Shark | *C. milii* | AFM87716.1 |
| *Canis lupus* | Wolf | *C.lupus* | P63113.2 |
| *Caretta caretta* | loggerhead | *C.caretta* | P56208.2 |
| *Clonorchis sinensis* | Chinese liver fluke | *C.sinensis* | AAM18464.1 |
| *Danio rerio* | zebra fish | *D.rerio* | AAH56727.1 |
| *Equus burchellii* | Plains Zebra | *E.burchellii* | P68083.2 |
| *Galeorhinus galeus* | school shark | *G.galeus* | P14397.2 |
| *Gallus gallus* | Chicken | *G.gallus* | P02197.4 |
| *Haloferax lucentense* | -- | *H.lucentense* | WP_004063165.1 |
| *Haloferax prahovense* | -- | *H.prahovense* | WP_008093645.1 |
| *Haloferax volcanii* | -- | *H.volcanii* | WP_004041552.1 |
| *Halogranum salarium* | -- | *H. salarium* | WP_009365573.1 |
| *Hemitriakis japanica* | Japanese tope shark | *H.japanica* | P14398.2 |
| *Heterodontus portusjacksoni* | Port Jackson Shark | *H.portusjacksoni* | P02206.2 |
| *Homo sapiens* | human | *H.sapiens* | AAH14547.1 |
| *Iguana iguana* | Green Iguana | *I.iguana* | ABN71515.1 |
| *Isoparorchis hypselobagri* | -- | *I.hypselobagri* | P80722.2 |
| *Macaca mulatta* | Rhesus Macaque | *M.mulatta* | XP_001081975.2 |
| *Mus musculus* | mouse | *M.musculus* | AAH25172.1 |
| *Mustelus antarcticus* | Gummy Shark | *M.antarcticus* | P14399.2 |
| *Nasonia vitripennis* | -- | *N.vitripennis* | XP_001608300.1 |
| *Ophelia bicornis* | -- | *O.bicornis* | AAX73248.1 |
| *Oryctolagus cuniculus* | Rabbit | *O.cuniculus* | P02170.2 |
| *Pan troglodytes* | chimpanzees | *P.troglodytes* | XP_001156646.1 |
| *Paragonimus westermani* | -- | *P.westermani Mb1*  *P.westermani Mb2* | AAX11352.1  AAX11353.1 |
| *Paramphistomum epiclitum* | -- | *P.epiclitum* | AAG48877.1 |
| *Physeter catodon* | Sperm Whale | *P.catodon* | BAF03579.1 |
| *Pongo abelii* | Sumatran Orangutan | *P.abelii* | NP_001125556.1 |
| *Rattus norvegicus* | Rat | *R.norvegicus* | AAH70511.1 |
| *Salmo salar* | Atlantic Salmon, | *S.salar* | ACM09229.1 |
| *Schistosoma mansoni* | -- | *S.mansoni* | XP_002578632.1 |
| *Sus scrofa* | Feral Pig | *S.scrofa* | NP_999401.1 |
| *Tetraodon nigroviridis* | Green Spotted Puffer | *T.nigroviridis* | Q701N9.4 |
| *Trichinella spiralis* | pork worm | *T.spiralis* | XP_003374157.1 |

**Table S2 Residues at site 34 and 68 of wild-type *Cs*Mb and different mutants**

|  | Residues |  |
| --- | --- | --- |
| Mutant | Site 34 | Site 68 |
| Wild-type | Tyr | Tyr |
| Y34A | Ala | Tyr |
| Y68A | Tyr | Ala |
| Y34A/Y68A | Ala | Ala |
